# Novel roles of DNA glycosylases in neurodegenerative diseases and aging

**DOI:** 10.4103/NRR.NRR-D-24-01588

**Published:** 2025-04-29

**Authors:** Vinod Tiwari, Fivos Borbolis, Deborah L. Croteau, Konstantinos Palikaras, Vilhelm A. Bohr

**Affiliations:** Section on DNA Repair, National Institute on Aging, Baltimore, MD, USA; Department of Physiology, Medical School, National and Kapodistrian University of Athens, Athens, Greece; Center for Healthy Aging, University of Copenhagen, Copenhagen, Denmark; Computational Biology & Genomics Core, LGG, NIA, Baltimore, MD, USA; Department of Diabetes & Cancer Metabolism, Beckman Research Institute of City of Hope, Duarte, CA, USA

Numerous neurological disorders negatively impact the nervous system, either through loss of neurons or by disrupting the normal functioning of neural networks. These impairments manifest as cognitive defects, memory loss, behavioral abnormalities, and motor dysfunctions. Decades of research have significantly advanced our understanding of the pathophysiology underlying neurodegenerative diseases, including Alzheimer’s disease (AD), Parkinson’s disease, amyotrophic lateral sclerosis, and others. Loss of dopaminergic neurons in the substantia nigra pars compacta with motor function defect is clinically associated with Parkinson’s disease, whereas the accumulation of amyloid-β plaques and tau neurofibrillary tangles is a main pathological hallmark of AD. Beyond these disease-specific mechanisms, key risk factors, such as aging, genomic stress, and mitochondrial dysfunction contribute broadly to the onset and progression of various neurological disorders (Tiwari and Wilson, 2019; Wilson et al., 2023). However, it is still unclear how these events promote the onset of pathological phenotypes and eventually lead to neuronal cell death. The DNA base excision repair (BER) pathway plays a vital role in the maintenance of genome stability and safeguarding human health by repairing endogenous and exogenous oxidative DNA damage. The BER pathway begins with a glycosylase enzyme that recognizes and removes the damaged base, creating an apurinic/apyrimidinic site. In the next step, an apurinic/apyrimidinic endonuclease cleaves the DNA backbone at the apurinic/apyrimidinic site, generating a single-strand break. This is followed by gap filling performed by DNA polymerase β, which inserts the correct nucleotide. Finally, DNA ligase seals the nick, restoring the integrity of the DNA strand.

The objective of our recent study (Tiwari et al., 2024) was to investigate how the loss of DNA glycosylases may contribute to the pathogenesis of neurological disease, utilizing a simple *Caenorhabditis (C*.*) elegans* model of human tauopathy. *C*. *elegans* constitutes a widely popular model organism for studying tauopathy and AD, with many studies reporting findings that are consistent with those observed in higher organisms. This underscores its relevance as a robust and translational model for understanding the molecular mechanisms underlying neurodegenerative pathologies.

Currently, there are conflicting perspectives on the role of dysfunctional DNA repair in driving premature aging diseases, and broad-spectrum neurological disorders, including AD. Evidence from our work and that of others has demonstrated that AD patients exhibit significant deficiencies in the BER pathway (Weissman et al., 2007; Jacob et al., 2013; Sykora et al., 2015). Notably, a deficiency in DNA polymerase β, an enzyme crucial for BER, has been shown to exacerbate the AD phenotype in an AD mouse model, underscoring the pivotal role of DNA repair in mitigating disease progression (Weissman et al., 2007; Sykora et al., 2015; Misiak et al., 2017). Recently, several studies, including our own, have shown that a deficiency in a DNA glycosylase, the damage recognition enzyme of the BER pathway, can protect mice and nematodes from DNA damage. This unexpected finding suggests that DNA glycosylase deficiency may confer neuroprotection, highlighting the complex and context-dependent role of BER enzymes in neural physiology (SenGupta et al., 2021).

To better understand this interesting dichotomy, we examined the involvement of DNA glycosylases in the determination of lifespan and healthspan in a tauopathy *C*. *elegans* model. As a simple organism, *C*. *elegans* possesses only two DNA glycosylases: the monofunctional UNG-1 DNA glycosylase and the bifunctional NTH-1 DNA glycosylase, making it an ideal system for studying their specific roles in this context. Tau-expressing nematodes exhibited reduced brood size, a defect that was reversed upon DNA glycosylase genetic ablation. Moreover, lifespan analyses in both wild-type and tau-expressing nematodes revealed that DNA glycosylase depletion did not significantly affect the median or maximum lifespan in wild-type animals. However, it significantly prolonged the shortened lifespan of tau-expressing worms. These findings suggest that DNA glycosylases contribute to tau-associated pathological features.

To investigate how DNA glycosylase deficiency affects neuroplasticity, we employed a chemotaxis assay for testing cognition. Our results revealed no significant memory deficits in either glycosylase mutant strain compared to wild-type animals. However, tau-expressing worms displayed severe cognitive impairment, consistent with previous studies (Fang et al., 2019), which was substantially ameliorated by depleting UNG-1 or NTH-1. Despite this protective effect, loss of either enzyme increased tau aggregation in aged worms compared to control animals. Given that oxidative stress increases with age, damaging macromolecules, such as DNA, this can ultimately lead to neuronal loss and exacerbate neurological disorders. Consequently, we assessed the lifespan of these worms under oxidative stress to better understand the long-term effect of DNA glycosylases on AD progression. As expected, tau-expressing worms showed a significantly shorter lifespan when exposed to the reactive oxygen generator menadione. Notably, UNG-1 depletion in tau-expressing worms further reduced their survival in response to oxidative stress, whereas NTH-1 deficient worms demonstrated enhanced lifespan compared to their respective counterparts. Our findings also suggest that there is a lack of compensatory mechanisms between UNG-1 and NTH-1, when either DNA glycosylase is depleted. Beyond DNA repair, we propose that these glycosylases may contribute to gene expression changes through epigenetic regulation.

Beyond oxidative stress, we also explored whether DNA glycosylases influence the lifespan of tau-expressing worms by contributing to the repair of lesions not typically handled by BER, such as cyclobutane pyrimidine dimers induced by ultraviolet C irradiation and crosslinks caused by cisplatin, both primarily handled by the nucleotide excision repair pathway. Tau-expressing nematodes displayed a marked reduction in lifespan upon ultraviolet C irradiation and cisplatin treatment, whereas wild-type and glycosylase-deficient animals showed no notable differences under the same conditions. Intriguingly, the deletion of DNA glycosylases conferred increased resistance to tau-expressing worms exposed to both ultraviolet C- and cisplatin-induced DNA damage. To uncover the molecular mechanisms underlying this neuroprotection and memory improvement, we conducted RNA sequencing analysis. Our results revealed 2817 differentially regulated genes in NTH-1 deficient worms, compared with just 44 in UNG-1 deficient worms relative to wild-type controls. Enrichment analysis of differentially expressed genes using WormBase identified significant gene ontology (GO) terms. None were found for UNG-1 deficient nematodes, possibly due to the small number of differentially expressed genes. However, NTH-1 deficient nematodes showed significant enrichment in 63 GO terms, including those related to DNA-directed RNA polymerase activity, splicing, actin dynamics, and muscle function. In tau-expressing worms, 34 significant GO terms were identified, primarily associated with muscle and actin function, immune system processes, calmodulin binding, and detoxification of cellular oxidants. When comparing tau-expressing DNA glycosylase mutants with wild-type animals, UNG-1 mutant worms showed enrichment in 15 GO terms, including defense responses, lytic vacuole, immune system processes, and axon injury response. In contrast, NTH-1 mutants exhibited a broader impact, with 78 enriched GO terms, covering recombinational repair, DNA polymerase activity, metabolic processes, cell death, splicing, and multiple muscle and actin-related terms. Notably, “response to axon injury” and “ABC-type transporter activity” were the only two GO terms common to both DNA glycosylase deficient tau-expressing nematode strains. To elaborate further on our findings, we compared our dataset with already existing human and mouse transcriptomic datasets. Five genes (*CNTN2*, *DLC2*, *PLCE1*, *SLC8A1*, and *COL21A1*) were found to overlap between tau-expressing worms and humans, while seven genes (*CNTN2*, *SOX2*, *ENPP2*, *HPGDS*, *MYO6*, *TUBB4A*, and *KL*) overlapped with mouse data. These results highlight a conserved role for NTH-1 in transcriptional regulation and strengthen its potential significance in neuroprotection and disease progression.

NTH-1 deficient nematodes exhibited elevated basal levels of reactive oxygen species (ROS), a known regulator of several stress response genes. To determine whether the observed protective effects of NTH-1 depletion in tau-expressing nematodes are mediated by ROS signaling, we treated the animals with the ROS scavenger N-acetyl-L-cysteine. While N-acetyl-L-cysteine supplementation displayed no effect on brood size, it partially reversed the lifespan extension observed in NTH-1 deficient tau-expressing worms under oxidative stress. Interestingly, lifespan was still prolonged in the presence of N-acetyl-L-cysteine, suggesting that the protective effects are at least partly regulated by ROS. DNA damage accumulation and impaired mitochondrial homeostasis are common features of many neurodegenerative diseases. Thus, we also assessed the status of DNA damage and mitochondrial membrane potential. Although the frequency of oxidized bases, as measured by 8-oxoG staining, was similar between wild-type and NTH-1 mutants, depletion of NTH-1 significantly reduced the elevated 8-oxoG levels observed in tau-expressing nematodes, indicating a protective effect. These findings were corroborated by a TUNEL assay, which revealed fewer single-stranded DNA breaks in NTH-1 deficient tauopathy worms. Furthermore, tau-expressing nematodes showed progressive loss of mitochondrial membrane potential with age, while NTH-1 deficiency preserved mitochondrial function in these animals. A similar protective effect was detected in UNG-1 deficient tau-expressing nematodes, though it diminished with aging. These results underscore the critical role of NTH-1 in the maintenance of mitochondrial function mitigating eventually tau-induced neurotoxicity.

Previous studies from our group in mouse models have shown that DNA polymerase β plays a protective role in AD progression (Misiak et al., 2017; Hou et al., 2018). In contrast, the present findings reveal that depleting DNA glycosylase activity can also be beneficial (**Figure 1**). Although we focused on a nematode model here, similar observations have been made in mice, such as the protective effect of DNA glycosylase depletion in a kidney infarct model (Ebrahimkhani et al., 2014).

**Figure 1:**
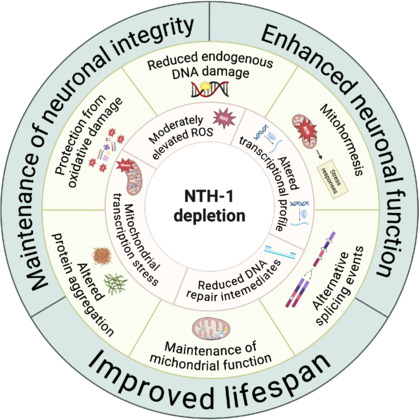
Multifaceted role of NTH-1 depletion in cellular homeostasis, neuronal health, and lifespan. NTH-1 depletion underscores its complex roles in maintaining neuronal integrity, enhancing neuronal function, and improving organismal lifespan. The inner ring highlights key molecular mechanisms impacted by NTH-1 depletion, including reduced endogenous DNA damage, moderately elevated reactive oxygen species (ROS), decreased DNA repair intermediates, and altered transcriptional profiles. These molecular changes lead to downstream effects (middle ring) such as protection from oxidative damage, reduced protein aggregation, alleviation of mitochondrial transcription stress, maintenance of mitochondrial function, mitohormesis, and modulation of alternative splicing events. Together, these processes (outer ring) contribute to improved neuronal integrity, enhanced neuronal function, and extended lifespan, showcasing the protective and adaptive potential of NTH-1 depletion in the context of neurodegenerative diseases and aging. Created with BioRender.com.

A key distinction between the two glycosylases studied here lies in their subcellular localization, which may influence their DNA repair functions and other cellular processes. NTH-1 is localized in both the nucleus and mitochondria (SenGupta et al., 2021), enabling it to address both genomic as well as mitochondrial DNA damage. In contrast, UNG-1 is confined to the nucleus, suggesting a more specialized role in preserving genomic integrity. These differences in localization could explain the distinct effects of each DNA glycosylase on neuronal health and AD progression observed in the current study.

Taken together, these findings highlight the context-dependent nature of DNA repair enzymes in disease progression, influenced by the stage of the repair process affected, the specific subcellular compartments targeted, and the potential non-canonical functions of the DNA repair enzymes. These facts underscore the complexity of DNA repair pathways and their diverse roles in the maintenance of tissue integrity and organismal health.

In conclusion, this study underscores the critical role of excessive DNA damage and the complexity of DNA repair mechanisms in the progression of neurological diseases. While preventing DNA damage and enhancing DNA repair are traditionally considered therapeutic strategies, our findings reveal that selective modulation of DNA repair pathways could offer novel and promising therapeutic interventions. Specifically, we observed that deficiency in DNA glycosylases can mitigate the harmful buildup of DNA repair intermediates, as the BER pathway is not initiated. These findings underline the potential of targeting specific components of DNA repair to tackle tau-mediated pathology.

Furthermore, this article and another study (Bordin et al., 2021) suggest that DNA glycosylases also participate in transcription-associated BER and possess a regulatory role in nuclear gene expression. These findings underscore the need for further investigation into the role of DNA repair proteins in transcription regulation and modulation. Beyond their repair functions, DNA glycosylases may modulate the transcriptional response of genes through mechanisms involving DNA methylation and epigenetic regulation. Another key area of interest highlighted in this study is the regulation of the spliceosome. Spliceosomes, large ribonucleoprotein complexes, are essential for generating mature mRNA. Disruption of splicing is a hallmark of AD. Hence, interference in RNA metabolism through mRNA splicing, modulated by DNA damage recognition and repair of lesions, represents an intriguing and unexplored avenue for future studies. However, it is crucial to recognize that the response of human and animal cells to DNA damage induced by extrinsic stress can differ significantly. Thus, caution is needed especially when extrapolating findings from animal models to humans. Further studies focused on human systems are essential to deepen our understanding and establish stronger correlations.

*This work was supported by the National Institute on Aging (Nos. AG000723 and AG000578) (to VAB); the Fondation Santé (No. 19656), Greece 2.0, the National Recovery and Resilience Plan’s flagship program TAEDR-0535850, and the European Research Council (No. 101077374 – SynaptoMitophagy) (to KP).*

## References

[R1] Bordin DL, Lirussi L, Nilsen H (2021). Cellular response to endogenous DNA damage: DNA base modifications in gene expression regulation. DNA Repair (Amst).

[R2] Ebrahimkhani MR, Daneshmand A, Mazumder A, Allocca M, Calvo JA, Abolhassani N, Jhun I, Muthupalani S, Ayata C, Samson LD (2014). Aag-initiated base excision repair promotes ischemia reperfusion injury in liver, brain, and kidney. Proc Natl Acad Sci U S A.

[R3] Fang EF, Hou Y, Palikaras K, Adriaanse BA, Kerr JS, Yang B, Lautrup S, Hasan-Olive MM, Caponio D, Dan X, Rocktäschel P, Croteau DL, Akbari M, Greig NH, Fladby T, Nilsen H, Cader MZ, Mattson MP, Tavernarakis N, Bohr VA (2019). Mitophagy inhibits amyloid-β and tau pathology and reverses cognitive deficits in models of Alzheimer’s disease. Nat Neurosci.

[R4] Hou Y, Lautrup S, Cordonnier S, Wang Y, Croteau DL, Zavala E, Zhang Y, Moritoh K, O’Connell JF, Baptiste BA, Stevnsner TV, Mattson MP, Bohr VA (2018). NAD(+) supplementation normalizes key Alzheimer’s features and DNA damage responses in a new AD mouse model with introduced DNA repair deficiency. Proc Natl Acad Sci U S A.

[R5] Jacob KD, Noren Hooten N, Tadokoro T, Lohani A, Barnes J, Evans MK (2013). Alzheimer’s disease-associated polymorphisms in human OGG1 alter catalytic activity and sensitize cells to DNA damage. Free Radic Biol Med.

[R6] Misiak M, Vergara Greeno R, Baptiste BA, Sykora P, Liu D, Cordonnier S, Fang EF, Croteau DL, Mattson MP, Bohr VA (2017). DNA polymerase β decrement triggers death of olfactory bulb cells and impairs olfaction in a mouse model of Alzheimer’s disease. Aging Cell.

[R7] SenGupta T, Palikaras K, Esbensen YQ, Konstantinidis G, Galindo FJN, Achanta K, Kassahun H, Stavgiannoudaki I, Bohr VA, Akbari M, Gaare J, Tzoulis C, Tavernarakis N, Nilsen H (2021). Base excision repair causes age-dependent accumulation of single-stranded DNA breaks that contribute to Parkinson disease pathology. Cell Rep.

[R8] Sykora P, Misiak M, Wang Y, Ghosh S, Leandro GS, Liu D, Tian J, Baptiste BA, Cong WN, Brenerman BM, Fang E, Becker KG, Hamilton RJ, Chigurupati S, Zhang Y, Egan JM, Croteau DL, Wilson DM, Mattson MP, Bohr VA (2015). DNA polymerase β deficiency leads to neurodegeneration and exacerbates Alzheimer disease phenotypes. Nucleic Acids Res.

[R9] Tiwari V, Wilson DM (2019). DNA damage and associated DNA repair defects in disease and premature aging. Am J Hum Genet.

[R10] Tiwari V, Buvarp E, Borbolis F, Puligilla C, Croteau DL, Palikaras K, Bohr VA (2024). Loss of DNA glycosylases improves health and cognitive function in a C. elegans model of human tauopathy. Nucleic Acids Res.

[R11] Weissman L, Jo DG, Sørensen MM, de Souza-Pinto NC, Markesbery WR, Mattson MP, Bohr VA (2007). Defective DNA base excision repair in brain from individuals with Alzheimer’s disease and amnestic mild cognitive impairment. Nucleic Acids Res.

[R12] Wilson DM, Cookson MR, Van Den Bosch L, Zetterberg H, Holtzman DM, Dewachter I (2023). Hallmarks of neurodegenerative diseases. Cell.

